# Does organic farming enhance biodiversity in Mediterranean vineyards? A case study with bats and arachnids

**DOI:** 10.1016/j.agee.2017.08.012

**Published:** 2017-11-01

**Authors:** Jérémy S.P. Froidevaux, Bastien Louboutin, Gareth Jones

**Affiliations:** a University of Bristol, School of Biological Sciences, Life Sciences Building, 24 Tyndall Avenue, BS8 1TQ Bristol, United Kingdom; b Office pour les insectes et leur environnement (Opie), Antenne Languedoc-Roussillon, 755 avenue du Campus Agropolis, 34 988 Montferrier-sur-Lez, France

**Keywords:** Acoustic sampling, Agriculture, Agri-environment schemes (AESs), Conservation, Landscape complexity

## Abstract

The effectiveness of organic farming for promoting biodiversity has been widely documented, yet most studies have been undertaken in temperate agroecosystems with a focus on birds, insects and plants. Despite the Mediterranean basin being a biodiversity hotspot for conservation priorities, the potential benefits of organic farming for biodiversity there has received little attention. Here, we assessed the effect of farming system, landscape characteristics and habitat structure on biodiversity in Mediterranean vineyards using two taxa with different functional traits (in terms of mobility, dispersal ability and home range size): bats and arachnids. We also tested the “intermediate landscape-complexity” hypothesis, which predicts that local conservation measures have greatest success in landscapes of intermediate complexity. Our study design involved pairs of matched organic and conventional vineyard plots in the south of France situated along a landscape complexity gradient. Abundance of arachnids were higher in organic vineyards, although arachnid species richness was positively associated with the amount of ground vegetation cover. Organic farming was ineffective on its own to enhance bat activity and species richness regardless of the landscape context. Rather, our results suggested that landscape features were more important for bats than vineyard management, with significantly higher bat activity recorded on vineyard plots located at close proximity to hedgerows and rivers. When designing conservation strategies in Mediterranean farmlands, we strongly recommend the implementation of a multi-scale approach to assure benefits for a wide range of species.

## Highlights

• Organic farming promotes arachnids but is ineffective on its own for bats in Mediterranean vineyards.
• Agri-environment schemes are not most effective for bats in landscapes of intermediate complexity.
• Bat activity was positively associated with proximity to hedgerows and rivers.
• A multi-scale approach is required to design adequate conservation strategies.

## 1 Introduction

Over the last 30 years, policies of the European Union (EU) have progressively evolved to try halting the dramatic loss of biodiversity that was associated to agricultural expansion and intensification (Henle et al., 2008, Pe’er et al., 2014). While the EU – with its Common Agricultural Policy (CAP) – has encouraged intensive and productive farming to ensure food security, problems of declining biodiversity were first addressed by the EU in 1985 by providing several measures for environmental protection to member states, and then during the 1992 CAP reform by developing and promoting Agri-Environmental Schemes (AESs) (Kleijn and Sutherland, 2003). This incentive system aims to counteract the negative effects of intensive agriculture by providing financial compensation to farmers that adopt environmentally-friendly farming approaches. AESs have become a key EU policy which aim to enhance biodiversity and ecosystem services in farmland (Whittingham, 2011) and represent the most expensive conservation programme implemented in Europe (Batáry et al., 2015): the EU will have allocated nearly 23 billion euros to AESs between 2014 and 2020 (European Parliament, 2016).

Support for conversion to organic farming is one of the main agri-environment schemes proposed to farmers. In 2015, farmlands under organic management represented 6.2% of utilised agricultural area in Europe (EU-28), comprising 11.1 million hectares, compared with 9.2 million hectares in 2010 (Eurostat, 2016). Due to the wildlife-friendly management implemented in organic farming (e.g., non-use of synthetic chemical pesticides and input fertilizers, low pressure on land-use) and its positive influence on landscape heterogeneity and complexity (Norton et al., 2009), organic farming would seem to be favourable for a range of taxa (Hole et al., 2005). However, several studies emphasize that the effects of organic farming on biodiversity are species-specific (Fuller et al., 2005) and most importantly, are dependent on the scale considered (Gabriel et al., 2010) and the landscape context (Batáry et al., 2011, Bengtsson et al., 2005, Tuck et al., 2014). Regarding the latter, the “intermediate landscape-complexity” hypothesis has been proposed to explain this pattern (Tscharntke et al., 2005; Tscharntke et al., 2012; Concepción et al., 2008). It stipulates that the effectiveness of organic farming would be higher in landscapes with intermediate level of complexity given that (i) extremely simplified landscapes are devoid of population sources and therefore do not allow possible re-colonisation; and (ii) the implementation of local conservation measure in more complex landscapes does not increase the species pool which is already high because of the complexity of the landscape (but see Allouche et al., 2012).

The effects of organic farming system on biodiversity have been, however, mainly investigated on birds, plants and insects in temperate grasslands and crops. Consequently, general results and ensuing recommendations may be not applicable to other taxa and other agricultural systems, especially those located in different bioclimatic regions (Tuck et al., 2014). Thus, there is a crucial need to re-assess the effectiveness of organic farming in non-temperate agroecosystems, especially in those located within biodiversity hotspots for conservation priorities (Myers et al., 2000), and to investigate the role of landscape characteristics in such systems.

Despite their roles as bioindicators and in pest suppression in agricultural areas (Jones et al., 2009, Boyles et al., 2011), insectivorous bats have been overlooked in studies assessing the effects of different agricultural management practices (Park, 2015). In Europe, little information is available for the Mediterranean basin, yet the area supports the highest bat species richness (Rebelo et al., 2010). In fact, only two studies reported the benefit of low-intensive management on bat activity and richness and these were restricted to olive groves (Davy et al., 2007, Herrera et al., 2015). While vineyards represent one of the main crop systems in several parts of the Mediterranean basin (e.g., in France 10.4% of lands located in the Mediterranean bioclimatic area are covered by vineyards), evidence on how bats may be affected by farm and landscape management in vineyard-dominated landscapes is lacking. At the farm scale, we could expect that organic farming would harbour more insect prey (Wickramasinghe et al., 2004), thus enhancing bat activity and species richness over organic fields (Wickramasinghe et al., 2003). At a broader scale, the presence of other foraging habitats (e.g., water bodies, forests) and roost sites (e.g., trees with cavities, man-made structures) are very likely to influence bat habitat use in vineyards (Rainho and Palmeirim, 2011). Given that populations of bats showed substantial declines during the second part of the 20th century partly due to the loss of foraging and commuting habitats within the agricultural matrix (Hutson and Mickleburgh, 2001), it is important to better understand bat-habitat relationships in vineyards to provide evidence-based conservation actions in these extensive habitats.

In this study, we aimed to determine whether organic farming is an efficient measure for enhancing bat activity and richness in Mediterranean vineyards. Our first objective was to disentangle the effect of landscape characteristics, farming system (organic vs. conventional) and vineyard structure on bat activity and species richness in order to provide adequate management recommendations. Given that bats are highly mobile and therefore capable to move across the landscape, we tested whether landscape features would be the main driver of bat activity and species richness in comparison to species with low-mobility which we hypothesized would be mainly affected by local management (Gonthier et al., 2014). We therefore used arachnids in addition to bats as biological models to test these hypotheses. Arachnids have a lower dispersal ability and home range size but like bats they occupy high trophic levels and may play a role in the suppression of pest populations (Marc et al., 1999), especially in vineyards (Emerit, 2011a, Drieu and Rusch, 2017). Furthermore, they may also be a good bioindicator taxon given their sensitivity to ecological change (Pearce and Venier, 2006, Gerlach et al., 2013). Our second objective was to investigate to role of landscape complexity in moderating the effect of organic farming on bats. We tested the “intermediate landscape-complexity” hypothesis (Tscharntke et al., 2012) using bat activity and species richness data that were collected along a gradient of landscape complexity.

## 2 Material and methods

### 2.1 Study area and site selection

**Fig. 1:**
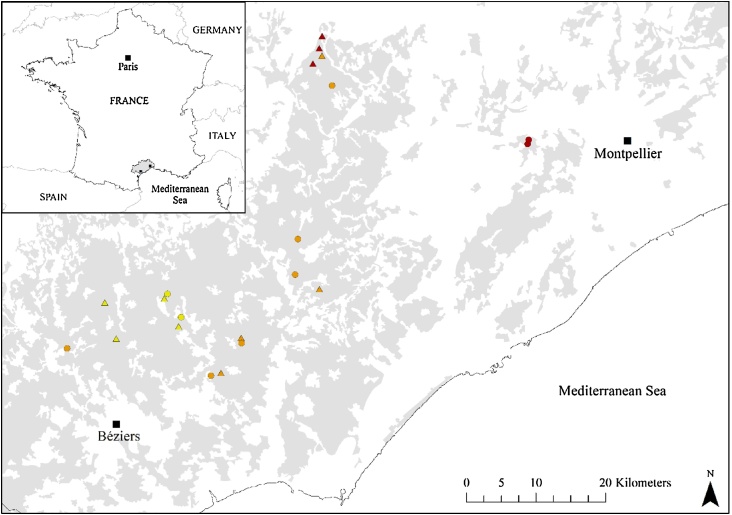
Location of the 21 paired sites in South of France (Hérault County). Each symbol represents a pair. Arachnids were sampled in 11 pairs (triangles) while acoustic sampling of bats took place in each pair (circles and triangles) situated along a landscape complexity gradient. Cleared landscapes (yellow symbols): extremely simplified landscape with <1% of forest and semi-natural areas; Simple landscapes (orange symbols): 1–20% of forest and semi-natural areas; Complex landscapes (red symbols): >20% of forest and semi-natural areas (Tscharntke et al., 2012). Vineyard areas are represented in grey (© CORINE Land Cover 2006, code 221). (For interpretation of the references to colour in this figure legend, the reader is referred to the web version of this article.)

The study area was located in the south of France, in Hérault County (Languedoc-Roussillon region; the second largest wine-growing area in France) between Montpellier and Béziers (Fig. 1). This area has a Mediterranean climate with characteristic hot, dry summers (e.g., Montpellier: mean temperature and rainfall for June, July, August, and September: 22.3 °C and 40 mm, respectively; www.meteofrance.com). The landscape consists mainly of a mosaic of fragmented habitats with 16% of the land covered by vineyards (96,761 ha in 2009), representing half of the total utilised agricultural area in the county (Direction Départementale des Territoires et de la Mer de l’Hérault, 2011). The major part of the vineyard area is distributed in the south-west of the county (Fig. 1). Although no information about pesticide use was collected in situ, Mailly et al. (2017) reported that the Languedoc-Roussillon region was – at the national level – one of the top three areas where the use of insecticide spray is the most intense in France. This research area seems therefore ideal to investigate the effect of farming system on biodiversity given that strong contrasts in terms of pesticide use are expected between organic and conventional vineyards.

The experiment was undertaken at paired sites, involving matched conventional and organic vineyard plots. This experimental set-up permits to control for nightly variation of bat activity (Hayes, 1997). Within pairs, sites were separated by distances between 500 and 5000 m to ensure collecting independent data in a relatively similar landscape context. As local management and landscape context are not strictly independent (Gabriel et al., 2009), we applied several criteria to obtain adequate pairs by considering (i) at the plot scale: the area, altitude, slope, and aspect of the vineyard plots as well as the presence/absence of linear features (i.e., hedgerows and tree lines) at their boundaries; and (ii) at the landscape scale: the proportion of area covered by vineyards and the proportion of forest and semi-natural areas (Table S1). At a 3.0 km radius scale, which corresponds to the core sustenance zone of many bat species, ten pairs were located in simple landscapes (i.e., landscapes with intermediate level of complexity: 1–20% of forest and semi-natural areas) while six were situated in cleared landscapes (i.e., extremely simplified landscapes: < 1% of forest and semi-natural areas) and five in complex landscapes (>20% of forest and semi-natural areas) (Tscharntke et al., 2012). Though other parameters might be used to determine the level of landscape complexity (e.g., Fahrig et al., 2011), we restricted the definition of complexity to the proportion of forest and semi-natural areas given the high dominance of vineyards within the study area. Sampling sites were located in the centre of the vineyard plot and situated at least 25 m away from hedgerows (mean: 73 m; range: 25–170 m), 50 m apart from urban areas (mean: 853 m; range: 53–1630 m), and 500 m away from main rivers (mean: 7320 m; range: 757–16,662 m) as we expected a strong influence of water bodies on bat activity during the sampling period (Russo and Jones, 2003, Sirami et al., 2013, Cruz et al., 2016). As such, the effect of farming system was isolated from potential confounding effects.

### 2.2 Bat echolocation call recording and identification

Bat sampling took place in 21 paired sites (Fig. 1; 42 detector-nights in total) from 9th August to 2nd September 2015, only during dry and warm nights (minimum temperature at night >15 °C) with low wind speed (≤4 on Beaufort scale). Both juvenile and adult bats are active during this period of the year. We used two Song Meter SM2BAT recorders (sampling rate: 384 kHz; Wildlife Acoustics, Concord, USA) connected to SMX-US ultrasonic microphones mounted on poles 2 m above the ground to simultaneously record bat echolocation calls at each vineyard plot. Each pair was acoustically sampled one full night, from 30 min before sunset until 30 min after sunrise. We switched detectors between each survey night (i.e., the detector that recorded bat calls in the organic plot was installed the following night in the conventional one, and vice versa) to avoid any bias due to microphone characteristics. Temperature at dusk was monitored using data loggers RC-5 (accuracy: ± 0.5 °C; Elitech, London, UK).

As acoustic sampling does not allow to differentiate individual bats, we used bat activity (i.e., number of bat passes) as a surrogate of bat abundance. We defined a bat pass as a series of minimum two bat echolocation calls with pulse interval(s) < 1 s. We manually identified each bat pass to species level when possible using BatSound 4.1.4. (Pettersson Electronic, Sweden). Identification criteria were based on call characteristics (including social calls) provided by Russo and Jones (2002), Pfalzer and Kusch (2003), Obrist et al. (2004), and Barataud (2012). We could not confidently identify calls from *Myotis* spp. and *Plecotus* spp. to species and therefore identified these calls at genus level. Regarding pipistrelle bats, ambiguous calls with end frequency situated at 50 kHz were classified as *Pipistrellus pygmaeus-pipistrellus*. *Pipistrellus nathusii* and *Pipistrellus kuhlii* show extensive overlap in their frequency of maximum energy, so were grouped together, although it is likely that most individuals comprised *P. kuhlii*. Similarly, it was not always possible to differentiate calls of *Nyctalus noctula* from *Nyctalus leisleri*, and we therefore identified some bat passes as *Nyctalus* spp. We then grouped bats according to their echolocation range (Schnitzler and Kalko, 2001) into three guilds namely short-, mid- and long-range echolocators (SRE, MRE and LRE respectively; see Table 1 and Frey-Ehrenbold et al. (2013) for more details). Nightly activity of a given bat guild was calculated by summing the number of passes of each bat species, complex of species and genus that constitutes the guild. We also quantified bat foraging activity by counting the number of feeding buzzes (i.e., final approach of a bat towards prey, distinguishable by the structure of the calls emitted) present within a bat pass. Foraging activity was highly correlated with total bat activity (Spearman’s rank correlation, *r*_s_ = 0.78, *d.f.* = 40, *P* < 0.001) and was therefore disregarded for the analysis. Finally, as most of the echolocation calls recorded were attributed to the MRE guild (95.6% of the bat passes), we decided to focus our activity analysis only on this guild as well as on the three taxa that dominate the guild (i.e., *P. nathusii*/*kuhlii*, *P. pipistrellus* and *P. pygmaeus*).

**Table 1:** Guild- and species-specific bat activity (number of bat passes) in organic and conventional vineyards. Numbers in brackets correspond to the total number of feeding buzzes.

Taxa	Organic vineyard	Conventional vineyard	Total
Long-range echolocators (LRE)	16 (1)	18 (2)	34 (3)
* Eptesicus serotinus*	0	2	2
* Nyctalus leisleri*	10	11	21
* Nyctalus* spp.	6	5	11
Mid-range echolocators (MRE)	741 (73)	978 (111)	1719 (184)
* Hypsugo savii*	15	11	26
* Miniopterus schreibersii*	8	12	20
* Pipistrellus nathusii-kuhlii*	238	257	495
* Pipistrellus pipistrellus*	217	297	514
* Pipistrellus pygmaeus*	240	361	601
* Pipistrellus pygmaeus-pipistrellus*	23	40	63
Short-range echolocators (SRE)	22 (1)	23 (1)	45 (2)
* Myotis* spp.	18	21	39
* Plecotus* spp.	1	0	1
* Rhinolophus hipposideros*	3	2	5
Total bat activity	779 (75)	1019 (114)	1798 (189)

### 2.3 Sampling of arachnids

Spiders and harvestmen were sampled at 11 paired-sites, during three consecutive days in August 2015 (Fig. 1). We installed eight 0.5 L pitfall traps arranged in a 7 × 10 m rectangle around the bat detector location. Traps were filled with soapsuds to reduce water surface tension. Samples were stored in tubes filled with 70% ethanol to preserve specimens before identification. We pooled the data from all 8 traps together at each site. Following Roberts (2009), Emerit (2011b), and Iorio and Delfosse (2016), adult spiders and harvestmen were identified where possible to species while juveniles to family. Online identification keys (e.g., www.araneae.unibe.ch) were also used to complete the identification.

### 2.4 Field survey and landscape data

To conduct field measurements on vineyard structure, we delimited a stand of 15 × 15 m aligned to the vine rows around each sampling site where the bat detector was installed. Within this, we collected a range of structural variables including the height of vine rows, distance between rows, ground vegetation cover, and ground vegetation height. For increased precision, information on ground vegetation were assessed in each quarter of the stand (7.5 × 7.5 m). Observer bias on the estimation of ground vegetation cover was minimal as the same person (lead author) collected all the data.

Arachnids, and especially bats are not restricted to vineyard plots, therefore the surrounding landscape may have a strong influence on their presence and abundance. To extract landscape characteristics at the most relevant spatial scales, we created four buffers (1.0, 2.0, 3.0 and 4.0 km radii) around the sampling sites using ArcGIS Desktop v10 (ESRI, Redlands, Canada). Smaller spatial scales were not taken into account as vineyards represent by far the most dominant habitat at such scales (∼92% at 0.5 km radius; Table S1). Within each buffer, we calculated the proportion of urban areas, arable land, vineyards, orchards, other agricultural areas, mixed and deciduous forests, coniferous forests, semi-natural areas, and freshwater surface using CORINE Land Cover data 2006 supplied by the European Environment Agency (www.eea.europa.eu; Table S2). We then incorporated these nine land classes within Fragstats 4.2. (McGarigal et al., 2002) to quantify the fragmentation, heterogeneity and diversity of the landscapes using the mean patch area, the patch richness density, and the Shannon’s diversity index respectively (see Froidevaux et al. (2017) for more details). Though no street lights were located at close vicinity to the sampling sites, we calculated at each spatial scale the amount of artificial light at night (nanowatts/cm^2^/sr; Earth Observation Group, NOAA National Geophysical Data Centre; www.ngdc.noaa.gov) given its potential effect on bat activity (Rowse et al., 2016). Finally, as bats may make extensive use of linear features (Boughey et al., 2011) as well as water sites especially in areas with a Mediterranean climate (Cruz et al., 2016, Russo and Jones, 2003, Sirami et al., 2013), we calculated for each sampling site the distance to (i) the nearest linear feature (i.e., hedgerow or tree line) previously mapped using Google Earth 2015; (ii) the nearest main river crossing the study area (i.e., Hérault or Orb); and (iii) the nearest perennial watercourse that includes streams, irrigation channels and ditches (BDTOPO^®^ Hydrography 2016, National Geographic Institute, France). We deliberately distinguished the main rivers from other perennial watercourses in our analyses as we expected the former to have a stronger influence on bats due to higher insect productivity and larger riparian zone (Salvarina, 2016).

### 2.5 Statistical analysis

To disentangle the influence of landscape characteristics, farming system (organic vs. conventional) and vineyard structure on bats and arachnids, we carried out several statistical analyses. Firstly, we tested the spatial independence of the response variables (i.e., activity of MRE, *P. pipistrellus*, *P. pygmaeus*, *P. nathusii*/*kuhlii*; abundance of arachnids, spiders and harvestmen; bat and arachnid species richness) by performing a Mantel test; no spatial correlation was found (Table S3). Then, each independent variable was standardized (i.e., rescaled) to allow direct comparisons of effect sizes. We performed data exploration to detect possible non-linear relationships between the response and independent variables. In order to reduce the number of landscape variables, we assessed independently the relationships between the response variables and each landscape variable measured at different spatial scales using a series of Generalized Linear Mixed-Effect models (GLMMs; “lme4” package; Bates et al., 2015) with the appropriate distribution (Poisson or negative binomial family to handle overdispersion) and considering the pair as a random effect. Variables that showed statistical significance (*P*-values < 0.05) were retained for the final full model. However, when the same variable was significant at different spatial scales, we chose the scale in which the variable had the largest effect size. For each response variable, we built a final full model that includes the landscape variables as well as the variables describing vineyard structure and the type of farming system. Temperature at dusk was also added as a covariate for models on bats. We assessed multicollinearity with the variance inflation factors (VIF values <3). Depending on the nature of the relationship found between the response and independent variables during data exploration, we used either GLMMs when linear relationships were expected or GAMs when non-linear relationships were detected. Abundance, activity and species richness were count data, we therefore used either a Poisson distribution or a negative binomial distribution when overdispersion was found. In both model types, pair was considered as a random effect to (i) allow pairwise comparison between organic and conventional vineyards and (ii) take into account similarities of landscape characteristics surrounding each pair. We ranked and selected the most parsimonious model using the second order information criterion (*AICc*; *“*MuMIN” package; Bartoń, 2016). When several models were identified as equivalent (*ΔAICc <*2), we chose the one having the fewest number of parameters (Burnham and Anderson, 2002). Finally, we tested independently the “intermediate landscape-complexity” hypothesis using GLMMs as previously described. We assessed the interaction between farming system and landscape complexity (factor composed of three levels: cleared, simple and complex landscapes) on MRE activity and bat species richness by performing least-squares mean comparisons (“lsmeans” package; Lenth, 2016) on each pairwise combinations while correcting for multiple comparisons (Tukey-Kramer method). All analyses were performed with R 3.3.2 (R Development Core Team, 2015).

## 3 Results

### 3.1 Bat and arachnid sampling

We recorded a total of 1798 bat passes within 42 vineyard plots (21 organic, 21 conventional) that belonged to 10 taxa (Table 1). The *Pipistrellus* genus dominated the bat assemblage with 1673 passes detected (93% of the bat activity). Amongst the pipistrelle species, *P. pygmaeus* was detected most frequently, followed by *P. pipistrellus* and then *P. nathusii*/*kuhlii*. At the guild level, SRE and LRE represented a fractional part of the activity recorded with only 45 (2.5%) and 34 (1.9%) bat passes, respectively. Nevertheless, we were able to detect some elusive bats such as *Rhinolophus hipposideros* and *Plecotus* spp. A total of 167 arachnid individuals were trapped in 22 vineyard plots (11 organic, 11 conventional) including 114 spiders and 53 harvestmen (Table S4). From this dataset we identified 21 species of spiders with 8 families represented and 3 species of harvestmen with 2 families. Individuals in the families Phalangiidae (harvestmen) and Gnaphosidae (ground spiders) were the most abundant, followed by spiders in the Zodariidae (ant spiders) and Lycosidae (wolf spiders). At the genus level, *Zelotes* spp. and *Zodarion* spp. dominated the spider assemblage with 25 individuals each, followed by *Pardosa* spp. and *Gnaphosa* spp. with 18 and 16 individuals, respectively.

### 3.2 Factors affecting bats and arachnids in vineyards

**Fig. 2:**
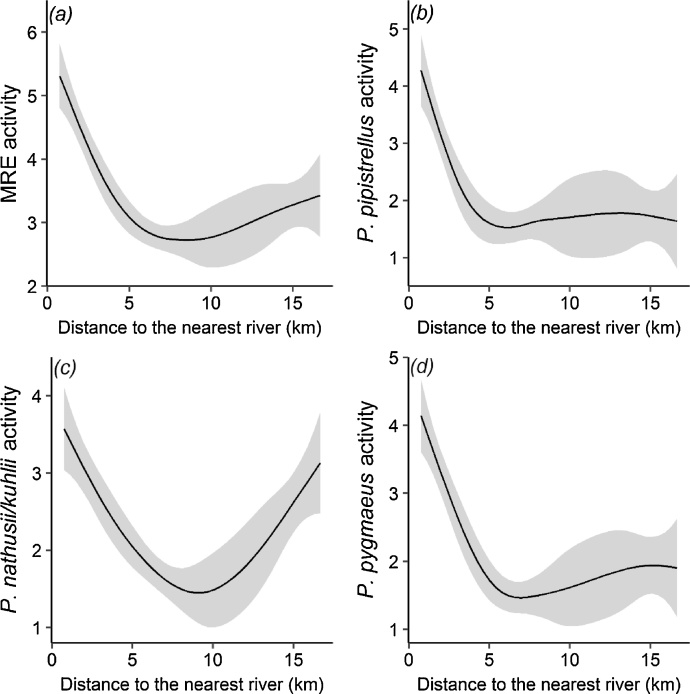
Non-linear relationship predicted by the most parsimonious GAMs (see Table 2) between the activity of (a) mid-range echolocator bats (MRE guild); (b) *Pipistrellus pipistrellus*; (c) *Pipistrellus nathusii*/*kuhlii*; and (d) *Pipistrellus pygmaeus* and the distance to the nearest river. Model predictions are represented by the black solid lines with 95% confidence interval indicated in grey.

**Fig. 3:**
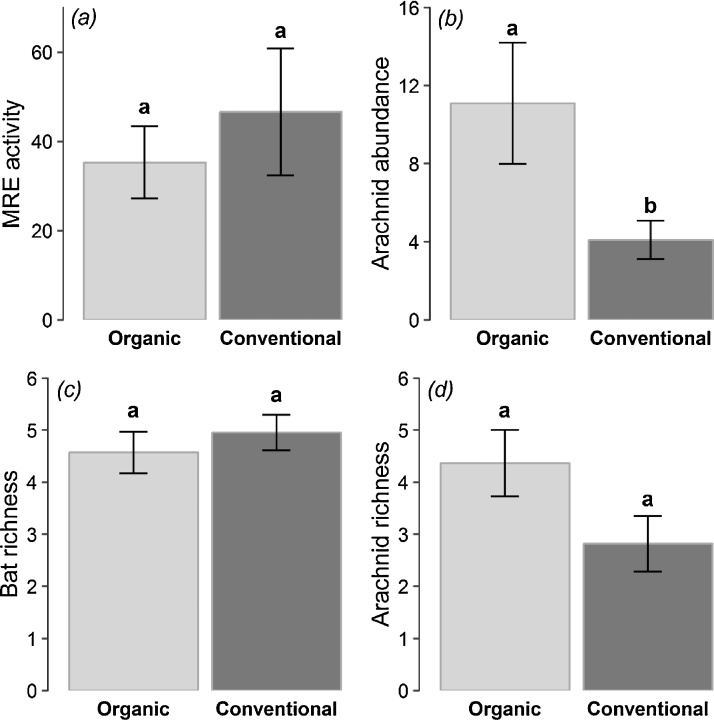
Mean (±SE) number of (a) bat passes of mid-range echolocator bats (MRE guild); (b) individuals of arachnids (spiders and harvestmen); (c) bat species; and (d) arachnid species in paired organic and conventional vineyards. Superscripts a and b are used to identify statistically significance differences between the two treatments (see Table 2).

**Fig. 4:**
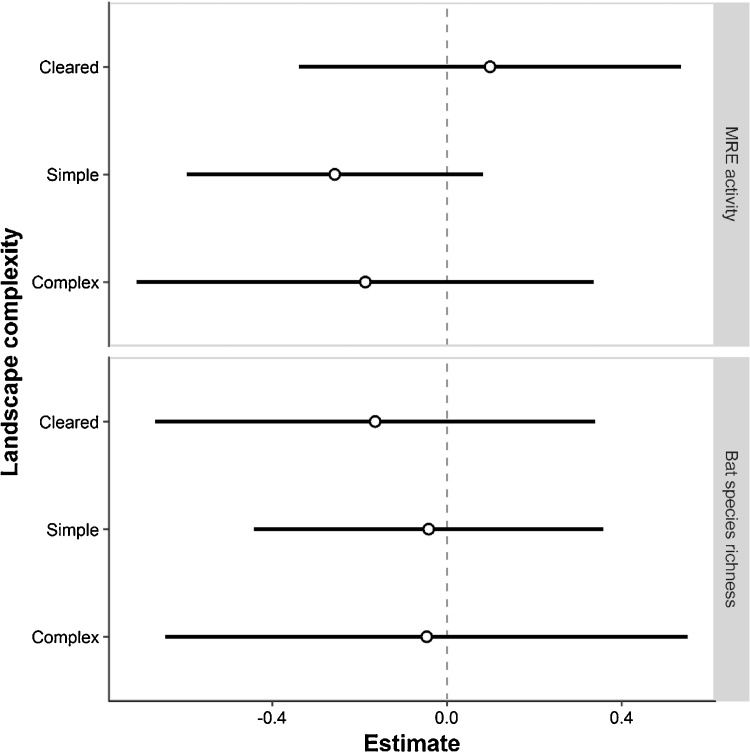
Effects of farming system (organic vs. conventional) on the activity of mid-range echolocator bats (MRE guild) and bat species richness in a gradient of landscape complexity at 3.0 km radius scale. Estimates and associated 95% confidence intervals arising from the pairwise comparisons of least-square means (“lsmeans” package) used to investigate variable interactions in GLMMs (see Section 2.5. Statistical analysis) are shown with white circles and black solid lines, respectively. Values on the right side of the dotted line suggest higher bat activity or species richness in organic vineyards. None of the comparisons are statistically significant (*P*-values >0.05). Cleared landscapes (6 pairs): extremely simplified landscape with <1% of forest and semi-natural areas; Simple landscapes (10 pairs): 1–20% of forest and semi-natural areas; Complex landscapes (5 pairs): >20% of forest and semi-natural areas (Tscharntke et al., 2012).

When assessing the relationship between bats and landscape characteristics, farming system (organic vs. conventional) and vineyard structure, our best models indicated relatively similar outputs for the different response variables (Table 2). Unlike distance to the nearest perennial stream, irrigation channel or ditch, distance to the nearest river (Hérault or Orb) was retained in all of the most parsimonious models on bat activity. These models suggest that bat activity decreases with increasing distance to the nearest river until a threshold distance after which activity tends to either stabilize or increase (Fig. 2). Our models also revealed the strong positive effect of temperature at dusk on both bat activity (excepted *P. nathusii*/*kuhlii*) and bat species richness as well as a significant negative effect of distance to the nearest linear feature on the activity of MRE, *P. pipistrellus* and *P. nathusii/kuhlii*, suggesting higher bat activity at sites near hedgerows or tree lines. Significant effects of vineyard structure were only highlighted in our models on *P. pygmaeus*, with a positive association found between its activity and vine row height and ground vegetation cover. Similarly, the proportion of urban areas at a large scale (2.0 km) had a significant and positive influence only on this species. We found, however, bat activity and species richness to be not significantly different between organic and conventional vineyard plots (Fig. 3), regardless of the level of complexity of the landscape (Fig. 4). Indeed, farming system was not selected by the most parsimonious models (Table 2, Table S5). Though bat activity was overall higher in conventional vineyards (Table 1), this pattern was skewed by two pairs (Fig. S6) in which conventional vineyards were slightly closer to river bodies (Hérault or Orb) than their organic counterparts.

**Table 2:** Standardized estimates (effect size) and standards errors (SE) of the variables present in the most parsimonious models (model selection based on *AICc*) relating to the effects of landscape characteristics, farming system (organic vs. conventional) and vineyard structure on bats and arachnids. MRE: mid-range echolocators.

Taxa	Response variable	Independent variable	Estimate (±SE)	Test statistic†	*p*
BAT	MRE activity^a^	Distance to the nearest river	*s*	84.66	***
	*Pseudo-R*^2^: 0.74	Distance to the nearest linear feature	−0.26 (±0.09)	−2.94	**
		Temperature at dusk	0.32 (±0.09)	3.40	***
	*P. pipistrellus* activity^a^	Distance to the nearest river	*s*	66.43	***
	*Pseudo-R*^2^: 0.74	Distance to the nearest linear feature	−0.31 (±0.11)	−2.70	**
		Temperature at dusk	0.61 (±0.12)	5.31	***
	*P. nathusii/kuhlii* activity^a^	Distance to the nearest river	*s*	45.91	^***^
	*Pseudo-R*^2^: 0.59	Distance to the nearest linear feature	−0.26 (± 0.11)	−2.34	*
	*P. pygmaeus* activity^a^	Distance to the nearest river	*s*	81.96	***
	*Pseudo-R*^2^: 0.82	Temperature at dusk	0.36 (±0.13)	2.84	**
		Vine row height	0.35 (±0.12)	3.08	**
		Ground vegetation cover	0.27 (±0.12)	2.29	*
		% of urban area within 2 km radius	0.35 (±0.10)	3.43	***
	Species richness^b^	Temperature at dusk	0.19 (±0.07)	2.68	^**^
	*Marginal R*^2^: 0.15				

ARACHNID	Total abundance^c^	Organic vs. conventional	0.99 (±0.18)	5.66	***
	*Marginal R*^2^: 0.25				
	Spider abundance^c^	Ground vegetation cover	0.42 (±0.11)	3.88	***
	*Marginal R*^2^: 0.30				
	Harvestmen abundance^c^	Organic vs. conventional	1.87 (±0.41)	4.52	***
	*Marginal R*^2^: 0.20				
	Species richness^b^	Ground vegetation cover	0.28 (±0.10)	2.73	**
	*Marginal R*^2^: 0.23				

MRE: mid-range echolocator bats. *s* represents the smooth term of GAMs. Pseudo-*R*^2^ are given for GAMs (Wood, 2006) while the marginal *R*^2^ (variance explained by the fixed effects only) are given for GLMMs (Nakagawa and Schielzeth, 2013).

a GAMs with a negative binomial distribution.

b GLMMs with a Poisson distribution.

c GLMMs with a negative binomial distribution.

† Chi-square value for the smooth terms of GAMs; Z value otherwise.

* *P* < 0.05.

** *P* < 0.01.

*** *P* < 0.001.

**Fig. 5:**
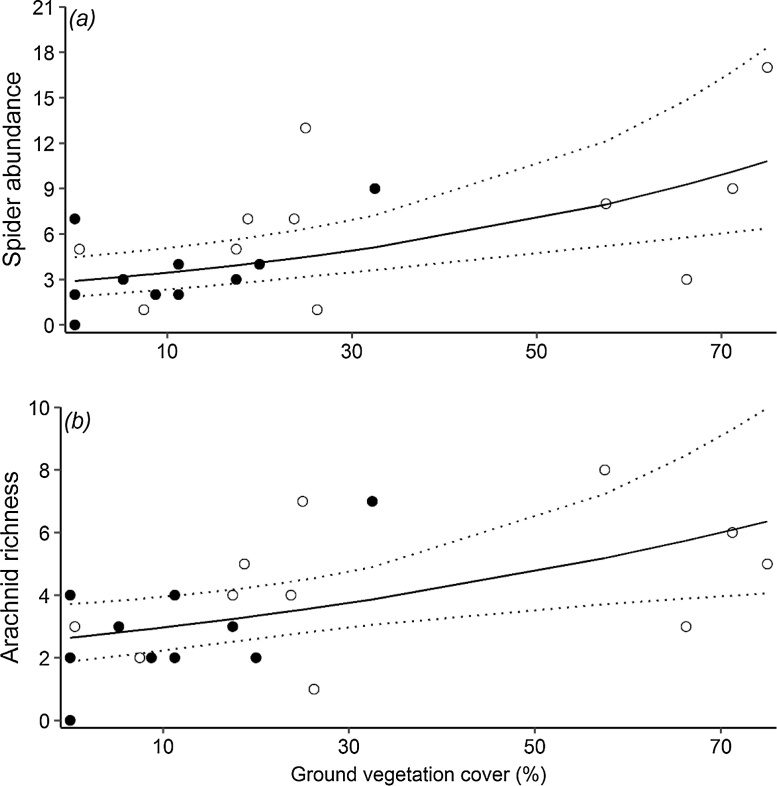
Effects of the proportion of ground vegetation cover on (a) spider abundance and (b) arachnid species richness in vineyards. Model predictions are represented by the black solid lines with 95% confidence interval indicated by the dotted lines. Open circles, organically-managed vineyards; filled circles: conventionally-managed vineyards.

When analysing the arachnid dataset, none of the landscape variables were selected in the most parsimonious models (Table 2). Our best model showed that, in comparison with organic vineyards, arachnids were significantly less abundant in conventional ones (Fig. 3). However, though the number of harvestmen individuals was also found to be significantly higher in organic vineyards, our best model indicated that spider abundance was significantly and positively related to the proportion of ground vegetation cover (Fig. 5). Regarding arachnid species richness, we did not find any statistical evidence that organic vineyards support greater numbers of arachnid species although overall fewer species were found in conventional vineyards (Fig. 3; Table S4). As with spider abundance, we found a significant and positive relationship between arachnid richness and the proportion of ground vegetation cover (Fig. 5). It is important to point out that the proportion of ground vegetation cover was significantly higher on organic than conventional vineyards (Paired *t*-test, *t* = 3.56, *d.f.* = 10, *P* < 0.01).

## 4 Discussion

The influence of agricultural management on biodiversity in Mediterranean agroecosystems has been poorly documented (Tuck et al., 2014), yet areas with Mediterranean climate are identified as biodiversity hotspots for conservation priorities (Myers et al., 2000). This study provides empirical evidence of the influence of landscape characteristics, farming system (organic vs. conventional) and habitat structure on two bioindicator taxa, bats and arachnids, that potentially play roles in the suppression of insect pest populations, in Mediterranean vineyards. Consistent with our hypothesis, our results suggest contrasting effects of local and landscape management on these taxa: while bats were mainly influenced by landscape characteristics, arachnids were affected by the management of vineyard plots. These findings highlight the necessity to implement a multi-scale approach when designing adequate conservation actions in farmland (Gonthier et al., 2014).

### 4.1 The effects of organic farming on bats and arachnids

Our main objective was to investigate the responses of two different taxa having different functional traits (mobility, dispersal ability and home range size) and the trends we document on arachnids fit with other empirical studies (see review of Prieto-Benitez and Mendez, 2011), even though our sample size was relatively limited and more focused on ground dwelling species than on the full assemblage. We found that organic vineyards enhance the abundance (Caprio et al., 2015) but not the species richness of arachnids (Schmidt et al., 2005, Bruggisser et al., 2010). The latter was indeed found to be positively influenced by the amount of ground vegetation cover. In fact, increasing ground vegetation cover may provide greater micro-habitat diversity as well as more favourable micro-climatic conditions, thus harbouring more prey (Costello and Daane, 1998, Paredes et al., 2013, Caprio et al., 2015). When analysing separately spider and harvestmen abundance, our results also indicated the importance of a high ground vegetation cover for spiders. An appropriate management of the vineyard ground cover therefore seems to provide a greater benefit for increasing species richness and spider abundance than does the low use of pesticides on its own. Nevertheless, this can also be seen as an indirect positive effect of organic farming as vegetation cover was significantly higher in organic vineyards. With respect to weed control, Bruggisser et al. (2010) emphasized that in Swiss vineyards mulching resulted in higher spider richness than mowing.

Regarding bats, there was no significant difference in bat activity at conventional and organic vineyards. Similarly, there was no clear evidence towards the benefit of organic farming on bat species richness. These results contradict previous studies on bats in temperate farmlands (Wickramasinghe et al., 2003, Fuller et al., 2005, MacDonald et al., 2012) which highlight the positive influence of organic farming on bat activity and richness. The lack of effect found in our study may arise from (i) the spray of insecticides in vineyards during early summer time – which was juridically mandatory regardless of the farming system – to control *Scaphoideus titanus* (Homoptera: Cicadellidae), the main vector of Flavescence dorée phytoplasma in European vineyards (Chuche and Thiéry, 2014); (ii) the low spatial aggregation and amount of organic vineyard plots within the landscapes (Rundlöf et al., 2008, Gabriel et al., 2010, Henckel et al., 2015) which may also rise some concerns regarding potential pesticide contamination of organic vineyards from adjacent conventional ones; (iii) the seasonal effect on bat habitat use (Heim et al., 2016) not investigated here as our acoustic dataset was collected during late summer only; (iv) the low level of use of vineyards by bats which may result from a preference for better foraging habitats such as water sites and remnant vegetation (Rambaldini and Brigham, 2011, Stahlschmidt et al., 2012, Sirami et al., 2013, Kelly et al., 2016); and (v) the selection of vineyard plots as we deliberately used information of the presence of linear features and other landscape characteristics to pair the vineyard plots and thus isolate the effect of farming system. Our findings, however, corroborate those of Long and Kurta (2014) who found no statistical differences in bat activity and species richness between organic and conventional apple orchards. Furthermore, Pocock and Jennings (2008) demonstrated that the presence of landscape features such as hedgerows within the farm is more important for bats than the low use of agrochemical inputs on its own. As the density of hedgerows was significantly higher on organic farms in the study of Fuller et al. (2005), it is then very likely that bats were enhanced by hedgerows rather than by the intensity of farming system. Similarly, Wickramasinghe et al. (2003) emphasized that part of the difference found in bat activity between organic and conventional farms may be attributed to hedgerow quality, with taller hedgerows observed in organic farms.

Despite sampling in different landscape contexts, our findings on bats are consistent regardless of the level of complexity of the landscapes and thus refute the *“*intermediate landscape-complexity*”* hypothesis (Tscharntke et al., 2005; Tscharntke et al., 2012; Concepción et al., 2008). This hypothesis proposes that the effectiveness of local conservation management within the agricultural matrix is higher in landscapes having 1–20% of semi-natural habitats (i.e., simple landscapes) compared to (i) cleared landscapes (<1% of non-crop habitats) as they are devoid of source populations; and (ii) complex landscapes (>20% semi-natural habitats) since the local diversity is high everywhere. Bats are highly mobile but their dispersal abilities in farmland may be constrained by the presence of acoustic landmarks such as hedgerows (Schnitzler and Kalko, 2001). Thus, the effectiveness of local conservation management on bats might also be determined by a gradient of landscape connectivity (Frey-Ehrenbold et al., 2013) in addition to complexity (but see Fahrig, 2013). In our study area, the structural connectivity was relatively low with a mean density of hedgerows and tree lines at 500 m radius around the vineyard plots <40 m/ha (39.67 ± 3.44 SE), which may explain the low level of activity recorded. Furthermore, though bats use different farmland habitats for foraging purposes (Russo and Jones, 2003), roost availability near potential foraging habitats may strongly influence the presence of bats and thus the success of conservation management (Rainho and Palmeirim, 2011). For instance, activity of bats such as *Pipistrellus* spp. roosting in man-made structures may depend on the presence of these particular roosts within the landscape. This is supported by our findings on *P. pygmaeus* as we found a positive relationship between its activity and the proportion of urban areas within a 2.0 km radius.

### 4.2 Landscape characteristics as drivers of bat activity

In areas that experience a Mediterranean climate, freshwater may be scarce during summer, and several studies emphasized the great benefits provided by riparian and other aquatic sites for bats, and recommended particular attention towards their management (Lisón and Calvo, 2011, Russo and Jones, 2003, Sirami et al., 2013). Indeed, bats use riparian sites for drinking but also for foraging due to the high abundance of insects emerging from the water and bankside vegetation (Fonderflick et al., 2015), especially during droughts when insect prey become scarcer in other habitats (Salvarina, 2016). In line with Cruz et al. (2016), our results show that habitats adjacent to major water sites (i.e., within a 5 km buffer; Fig. 3) support higher bat activity. The underlying key mechanisms behind this finding remain, however, to be tested. The two main hypotheses are namely (i) increased prey abundance due to the dispersal of insect prey from water sites and riparian vegetation to the surrounding habitats; and (ii) the selection of suitable roosts near important drinking and foraging sites to reduce commuting costs (Korine et al., 2016, Rainho and Palmeirim, 2011).

The great benefit of linear features on bats in farmlands has been widely documented (e.g., Boughey et al., 2011, Frey-Ehrenbold et al., 2013), yet little is known of their effects in vineyards. A recent study conducted in Californian vineyards suggested that maintaining remnant vegetation on the vineyard boundaries enhances bat activity (Kelly et al., 2016). The strong negative relationship found in our study between bat activity and distance to the nearest hedgerow or tree line supports this finding. As bats use these linear elements for foraging as well as commuting between foraging habitats and roosts (Limpens and Kapteyn, 1991), it is not surprising to have found higher activity near such important corridors for bats. Nevertheless, despite the extensive literature on the roles of green features on bats in agricultural landscapes, evidence-based information on their management are crucially lacking. Future research should focus on determining best management practices in order to maximize their benefits on bats.

### 4.3 Implications for conservation

Although management recommendations towards the enhancement of arachnid populations in vineyards have previously been highlighted (Bruggisser et al., 2010, Caprio et al., 2015), little information is available for promoting bat activity and species richness. Considering the severe population decline that bats encountered during the 20th century and their potential role in the suppression of insect pests in agricultural ecosystems, providing effective management recommendations that benefit bats in vineyards is needed urgently. Organic farming has been proposed as a key measure to counteract the negative effects of agricultural intensification on biodiversity, yet we found this measure to be ineffective on its own for enhancing bat activity and species richness in vineyards. Rather, our results suggest that conservation actions should focus on (i) the creation and maintenance of key landscape characteristics such as freshwater sites and linear features; and (ii) increasing roost availability within the landscape with, for instance, the installation of bat-boxes for pipistrelle bats (Flaquer et al., 2006). Although the creation of artificial wetlands might be considered in areas devoid of freshwater sites given their great value for bats in vineyards (Stahlschmidt et al., 2012, Sirami et al., 2013), priority should be given on the conservation and restoration of existing natural ones. Hedgerows and tree lines are of major importance for bats (Boughey et al., 2011, Frey-Ehrenbold et al., 2013) and may affect bat colony size at larger scales (Froidevaux et al., 2017), it is therefore essential to create a dense and connected network of these corridors to improve landscape permeability and thus facilitate access to suitable foraging habitats. As observed in temperate vineyards (Stahlschmidt et al., 2012), the bat assemblage recorded in vineyards was dominated by pipistrelle bats. However, though the effects of agricultural management may be species- and/or guild-specific (Park, 2015), we assume that short-range echolocator bats – which are subject of major conservation concerns given their high probability of extinction risk (Jones et al., 2003, Safi and Kerth, 2004) – are very likely to benefit from the creation of hedgerows due to their ecological and morphological adaptations to forage at close vicinity to vegetation (Schnitzler and Kalko, 2001).

## 5 Conclusion

The implementation of environmentally friendly farming systems, such as organic farming, in Mediterranean vineyards have contrasting outputs depending on the taxa of interest (Puig-Montserrat et al., 2017). Though an appropriate management of the vineyards at the plot scale may enhance low mobility species that have relatively small home range such as arachnids, a landscape-scale approach is required for higher mobility species like bats (Treitler et al., 2016). The management of vineyard plots under organic farming conditions alongside the maintenance of a high proportion of ground vegetation cover are the two main recommendations to favour arachnid biodiversity. Regarding bats, conservation actions should focus on increasing landscape connectivity through the creation of hedgerows, water accessibility with the restoration/creation of freshwater sites and roost availability with for instance the installation of bat-boxes. Considering the recent advances in molecular analysis, we finally encourage future research to (i) assess the diet of bats foraging over vineyards and evaluate the ecosystem services that bats may provide for this agroecosystem (Williams-Guillén et al., 2016); and (ii) investigate the exposure of bats to pesticides in agricultural landscapes dominated by different farming systems (organic, integrated and conventional farming).
