# Supplementary material for: Does organic farming enhance biodiversity in Mediterranean vineyards? A case study with bats and arachnids
Source: Agric Ecosyst Environ. 2017 Nov 1;249:112–22. doi: 10.1016/j.agee.2017.08.012 (PMC5614100; doi:10.1016/j.agee.2017.08.012)
Supplement: Supplementary file 1 [file mmc1.docx]

Supplementary Data

Does organic farming enhance biodiversity in Mediterranean vineyards? A case study with bats and arachnids.

Jérémy S. P. Froidevaux, Bastien Louboutin, and Gareth Jones

**Table S1**. Pairwise comparison of plot and landscape variables assessed during fieldwork preparation to obtain adequate pairs of organic and conventional vineyards. None of the comparisons are statistically significant (*P*-values > 0.05).

| **Scale** | **Variable** | **Organic vineyard**  mean (± SE) | **Conventional vineyard**  mean (± SE) | **Test**^a^ | ***P*** |
| --- | --- | --- | --- | --- | --- |
| Plot | Altitude (m a.s.l.) | 79.58 (± 9.31) | 83.2 (± 10.70) | Paired t-test | 0.44 |
|  | Slope (°) | 4.51 (± 0.82) | 3.77 (± 0.92) | Paired t-test | 0.31 |
|  | Aspect (°) | 181.22 (± 15.49) | 167.61 (± 15.26) | Paired t-test | 0.49 |
|  | Area (ha) | 2.43 (± 0.35) | 2.47 (± 0.31) | Paired t-test | 0.93 |
| Landscape^c^ | % of vineyard within 0.5 km radius buffer | 90.89 (± 3.19) | 92.22 (± 2.83) | Permutation test | 0.68 |
|  | % of forest and semi-natural habitats within 0.5 km radius buffer | 3.09 (± 1.63) | 5.02 (± 2.69) | Permutation test | 0.34 |
|  | % of vineyard within 1.0 km radius buffer | 81.85 (± 3.37) | 84.12 (± 3.41) | Paired t-test | 0.38 |
|  | % of forest and semi-natural habitats within 1.0 km radius buffer | 7.16 (± 2.47) | 7.77 (± 3.30) | Permutation test | 0.71 |
|  | % of vineyard within 2.0 km radius buffer | 73.43 (± 3.44) | 71.74 (± 4.06) | Paired t-test | 0.38 |
|  | % of forest and semi-natural habitats within 2.0 km radius buffer | 9.22 (± 2.86) | 12.03 (± 3.76) | Permutation test | 0.16 |
|  | % of vineyard within 3.0 km radius buffer | 69.61 (± 3.81) | 68.13 (± 4.56) | Paired t-test | 0.39 |
|  | % of forest and semi-natural habitats within 3.0 km radius buffer | 12.31 (± 3.32) | 14.96 (± 4.23) | Paired t-test^b^ | 0.67 |
|  | % of vineyard within 4.0 km radius buffer | 68.88 (± 4.05) | 67.27 (± 4.44) | Paired t-test | 0.19 |
|  | % of forest and semi-natural habitats within 4.0 km radius buffer | 14.01 (± 3.59) | 16.30 (± 4.33) | Permutation test | 0.11 |

^a^ Paired t-test when data were normally distributed; Permutation test otherwise (Monte Carlo permutation test for paired individual scores; “surveillance” package; 9999 permutations).

^b^ Data were log-transformed to achieve normality.

^c^ Landscape features extracted within a buffer of 0.5, 1.0, 2.0, 3.0 and 4.0 km radii around the sampling sites.

**Table S2.** Description of the reclassified CORINE Land Cover data 2006 (CLC) used to (i) characterize the different habitats surrounding the vineyard plots and (ii) perform the landscape analysis.

| **CLC code** | **CLC description** | **New code** | **New description** |
| --- | --- | --- | --- |
| 112 | Discontinuous urban fabric | 1 | Urban area |
| 121 | Industrial or commercial units | 1 | Urban areas |
| 131 | Mineral extraction sites | 1 | Urban areas |
| 142 | Sport and leisure facilities | 1 | Urban areas |
| 211 | Non-irrigated arable land | 2 | Arable lands |
| 221 | Vineyards | 3 | Vineyards |
| 222 | Fruit trees and berry plantations | 4 | Orchards |
| 223 | Olive groves | 4 | Orchards |
| 242 | Complex cultivation patterns | 5 | Other agricultural areas |
| 243 | Land principally occupied by agriculture, with significant areas of natural vegetation | 5 | Other agricultural areas |
| 311 | Broad-leaved forest | 6 | Mixed and deciduous forests |
| 312 | Coniferous forest | 7 | Coniferous forests |
| 313 | Mixed forest | 6 | Mixed and deciduous forests |
| 321 | Natural grasslands | 8 | Semi-natural areas |
| 323 | Sclerophyllous vegetation | 8 | Semi-natural areas |
| 324 | Transitional woodland-shrub | 8 | Semi-natural areas |
| 333 | Sparsely vegetated areas | 8 | Semi-natural areas |
| 511 | Water courses | 9 | Freshwater surface |
| 512 | Water bodies | 9 | Freshwater surface |

**Table S3.** Results of the Mantel test performed to investigate spatial correlation (*r*) of the response variables. No correlation was found (empirical *P*-values > 0.05; 9999 permutations).

| **Taxa** | **Response variable** | ***\|r\|*** | ***P*** |
| --- | --- | --- | --- |
| BAT | MRE^a^ activity | 0.05 | 0.64 |
|  | *Pipistrellus pipistrellus* activity | 0.06 | 0.77 |
|  | *Pipistrellus nathusii/kuhlii* activity | 0.09 | 0.92 |
|  | *Pipistrellus pygmaeus* activity | 0.02 | 0.33 |
|  | Species richness | 0.01 | 0.49 |
| ARACHNID | Arachnid abundance | 0.03 | 0.66 |
|  | Spider abundance | 0.01 | 0.42 |
|  | Harvestmen abundance | 0.07 | 0.91 |
|  | Species richness | 0.05 | 0.20 |

^a^ MRE: Mid-range echolocator bats.

**Table S4.**  Number of individuals of spiders and harvestmen collected in organic and conventional vineyards. Positive values are displayed in bold for a better visual comparison. The last row indicates the total species richness.

| **Order** | **Family** | **Species** | | **Organic vineyard** | **Conventional vineyard** | | **Total** | |  |
| --- | --- | --- | --- | --- | --- | --- | --- | --- | --- |
| Araneae | Agelenidae | *Eratigena agrestis* | | **5** | **1** | | 6 | |  |
| Araneae | Agelenidae | spp. | | 0 | **1** | | 1 | |  |
| Araneae | Gnaphosidae | *Gnaphosa alacris* | | **2** | **3** | | 5 | |  |
| Araneae | Gnaphosidae | *Gnaphosa spp.* | | **8** | **3** | | 11 | |  |
| Araneae | Gnaphosidae | *Nomisia aussereri* | | **2** | 0 | | 2 | |  |
| Araneae | Gnaphosidae | *Setaphis carmeli* | | **1** | 0 | | 1 | |  |
| Araneae | Gnaphosidae | *Trachyzelotes fuscipes* | | **1** | 0 | | 1 | |  |
| Araneae | Gnaphosidae | *Zelotes aeneus* | | **14** | **8** | | 22 | |  |
| Araneae | Gnaphosidae | *Zelotes callidus* | | **1** | 0 | | 1 | |  |
| Araneae | Gnaphosidae | *Zelotes tenuis* | | 0 | **1** | | 1 | |  |
| Araneae | Gnaphosidae | *Zelotes thorelli* | | **1** | 0 | | 1 | |  |
| Araneae | Lycosidae | *Hogna radiata* | | **1** | **1** | | 2 | |  |
| Araneae | Lycosidae | *Pardosa hortensis* | | **2** | **2** | | 4 | |  |
| Araneae | Lycosidae | *Pardosa occidentalis* | | **1** | 0 | | 1 | |  |
| Araneae | Lycosidae | *Pardosa proxima* | | **2** | **2** | | 4 | |  |
| Araneae | Lycosidae | *Pardosa spp.* | | **7** | **2** | | 9 | |  |
| Araneae | Lyniphiidae | *Agyneta pseudorurestris* | | **1** | 0 | | 1 | |  |
| Araneae | Lyniphiidae | spp. | | **2** | **1** | | 3 | |  |
| Araneae | Lyniphiidae | *Tenuiphantes tenuis* | | 0 | **1** | | 1 | |  |
| Araneae | Nemesiidae | *Nemesia* spp. | | 0 | **1** | | 1 | |  |
| Araneae | Philodromidae | spp. | | **3** | 0 | | 3 | |  |
| Araneae | Philodromidae | *Thanatus atratus* | | **2** | 0 | | 2 | |  |
| Araneae | Salticidae | *Aelurillus v-insignitus* | | 0 | **1** | | 1 | |  |
| Araneae | Salticidae | *Pellenes* spp. | | **2** | 0 | | 2 | |  |
| Araneae | Salticidae | spp. | | **1** | **1** | | 2 | |  |
| Araneae | Zodariidae | *Zodarion elegans* | | **14** | **6** | | 20 | |  |
| Araneae | Zodariidae | *Zodarion pseudoelegans* | | **1** | 0 | | 1 | |  |
| Araneae | Zodariidae | *Zodarion* spp. | | **1** | **3** | | 4 | |  |
| Opiliones | Phalangiidae | *Odiellus spinosus* | | **1** | 0 | | 1 | |  |
| Opiliones | Phalangiidae | *Odiellus troguloides* | | **6** | **1** | | 7 | |  |
| Opiliones | Phalangiidae | *Phalangium opilio* | | **3** | **2** | | 5 | |  |
| Opiliones | Phalangiidae | spp. | | **35** | **3** | | 38 | |  |
| Opiliones | Trogulidae | spp. | | **1** | **2** | | 3 | |  |
| **Total abundance** | | | 121 | | | 46 | | 167 | |
| **Total species richness** | | | 21 | | | 15 | | 25 | |

**Table S5.** Summary table of step-wise selection based on the *AICc* of GAMs and GLMMs used to investigate the relative importance of landscape characteristics, farming system (organic vs. conventional) and vineyard structure on bats and arachnids. Only the most parsimonious models are shown (*ΔAICc*<2), ranked by *AICc* values, with best models (i.e. models having the lowest number of variables) displayed in bold. *AICc* weight (*Wt*), cumulative weight (*Cum. Wt*) and *R*^2^ values are given for each model.

| Taxa | Response variable | Model | *K* | *AICc* | *ΔAICc* | *AICc Wt* | *Cum. Wt* | *R*^2a^ |
| --- | --- | --- | --- | --- | --- | --- | --- | --- |
| BAT | MRE activity^b^ | **Distance to linear feature + *s*(Distance to river) + Temperature** | **7** | **356.60** | **0.00** | **0.70** | **0.70** | **0.74** |
|  |  | Distance to linear feature + *s*(Distance to river) + Temperature + Farming system | 8 | 358.31 | 1.71 | 0.30 | 1.00 | 0.75 |
|  |  |  |  |  |  |  |  |  |
|  | *P. pipistrellus* activity^b^ | **Distance to linear feature + *s*(Distance to river) + Temperature** | **8** | **261.90** | **0.00** | **1.00** | **1.00** | **0.74** |
|  |  |  |  |  |  |  |  |  |
|  | *P. nathusii/kuhlii* activity^b^ | Distance to linear feature + *s*(Distance to river) + Farming system + Vine row height | 8 | 268.80 | 0.00 | 0.27 | 0.27 | 0.66 |
|  |  | Distance to linear feature + *s*(Distance to river) + Ground vegetation height | 7 | 269.75 | 0.95 | 0.17 | 0.44 | 0.62 |
|  |  | Distance to linear feature + *s*(Distance to river) + Vine row height | 7 | 269.67 | 0.87 | 0.17 | 0.61 | 0.62 |
|  |  | Distance to linear feature + *s*(Distance to river) + Temperature + Farming system + Vine row height | 9 | 269.97 | 1.17 | 0.15 | 0.76 | 0.68 |
|  |  | **Distance to linear feature + *s*(Distance to river)** | **6** | **270.26** | **1.46** | **0.13** | **0.89** | **0.59** |
|  |  | Distance to linear feature + *s*(Distance to river) + Farming system + Vine row height + Fragmentation at 3000 m | 9 | 270.56 | 1.76 | 0.11 | 1.00 | 0.68 |
|  |  |  |  |  |  |  |  |  |
|  | *P. pygmaeus* activity^b^ | ***s*(Distance to river) + Temperature + Vine row height + Ground vegetation cover + % of urban area at 2000 m** | **10** | **265.10** | **0.00** | **1** | **1.00** | **0.82** |
|  |  |  |  |  |  |  |  |  |
|  | Species richness^c^ | **Temperature** | **3** | **168.20** | **0.00** | **0.27** | **0.27** | **0.15** |
|  |  | Temperature + Distance between vine rows | 4 | 168.24 | 0.04 | 0.27 | 0.54 | 0.20 |
|  |  | Temperature + Ground vegetation height | 4 | 169.61 | 1.41 | 0.13 | 0.67 | 0.17 |
|  |  | Temperature + Ground vegetation cover + Distance between vine rows | 5 | 169.98 | 1.78 | 0.11 | 0.78 | 0.21 |
|  |  | Temperature + Ground vegetation cover | 4 | 169.99 | 1.79 | 0.11 | 0.89 | 0.16 |
|  |  | Temperature + Ground vegetation height + Distance between vine rows | 5 | 170.09 | 1.89 | 0.11 | 1.00 | 0.20 |
|  |  |  |  |  |  |  |  |  |
| ARACHNID | Total abundance^d^ | Farming system + % of semi-natural area at 4000 m | 5 | 133.30 | 0.00 | 0.39 | 0.39 | 0.46 |
|  |  | **Farming system** | **4** | **134.52** | **1.22** | **0.21** | **0.60** | **0.25** |
|  |  | Ground vegetation cover + % of semi-natural area at 4000 m + Distance between vine rows | 6 | 134.60 | 1.30 | 0.20 | 0.80 | 0.47 |
|  |  | Farming + Ground vegetation cover + % of semi-natural area at 4000 m | 6 | 134.71 | 1.41 | 0.20 | 1.00 | 0.51 |
|  |  |  |  |  |  |  |  |  |
|  | Spider abundance^d^ | **Ground vegetation cover** | **4** | **116.80** | **0.00** | **1.00** | **1.00** | **0.30** |
|  |  |  |  |  |  |  |  |  |
|  | Harvestmen abundance^d^ | **Farming system** | **4** | **86.00** | **0.00** | **0.30** | **0.30** | **0.20** |
|  |  | Farming system + Ground vegetation height | 5 | 86.39 | 0.39 | 0.25 | 0.55 | 0.15 |
|  |  | Farming system + Distance to linear feature | 5 | 87.38 | 1.38 | 0.15 | 0.70 | 0.19 |
|  |  | Ground vegetation cover + Distance between vine rows | 5 | 87.44 | 1.44 | 0.15 | 0.85 | 0.20 |
|  |  | Farming + Distance between vine rows | 5 | 87.49 | 1.49 | 0.15 | 1.00 | 0.17 |
|  |  |  |  |  |  |  |  |  |
|  | Species richness^c^ | **Ground vegetation cover** | **3** | **92.00** | **0.00** | **0.58** | **0.58** | **0.23** |
|  |  | Ground vegetation cover + Distance to linear feature | 4 | 92.64 | 0.64 | 0.42 | 1.00 | 0.30 |

^a^ Pseudo-*R*^2^ are given for GAMs (Wood, 2006) while the marginal *R*^2^ (variance explained by the fixed effects only) are given for GLMMs (Nakagawa and Schielzeth, 2013).

^b^ GAMs with a negative binomial distribution; ^c^ GLMMs with a Poisson distribution; ^d^ GLMMs with a negative binomial distribution.

*s* represents the smooth term of GAMs.

MRE: mid-range echolocators.


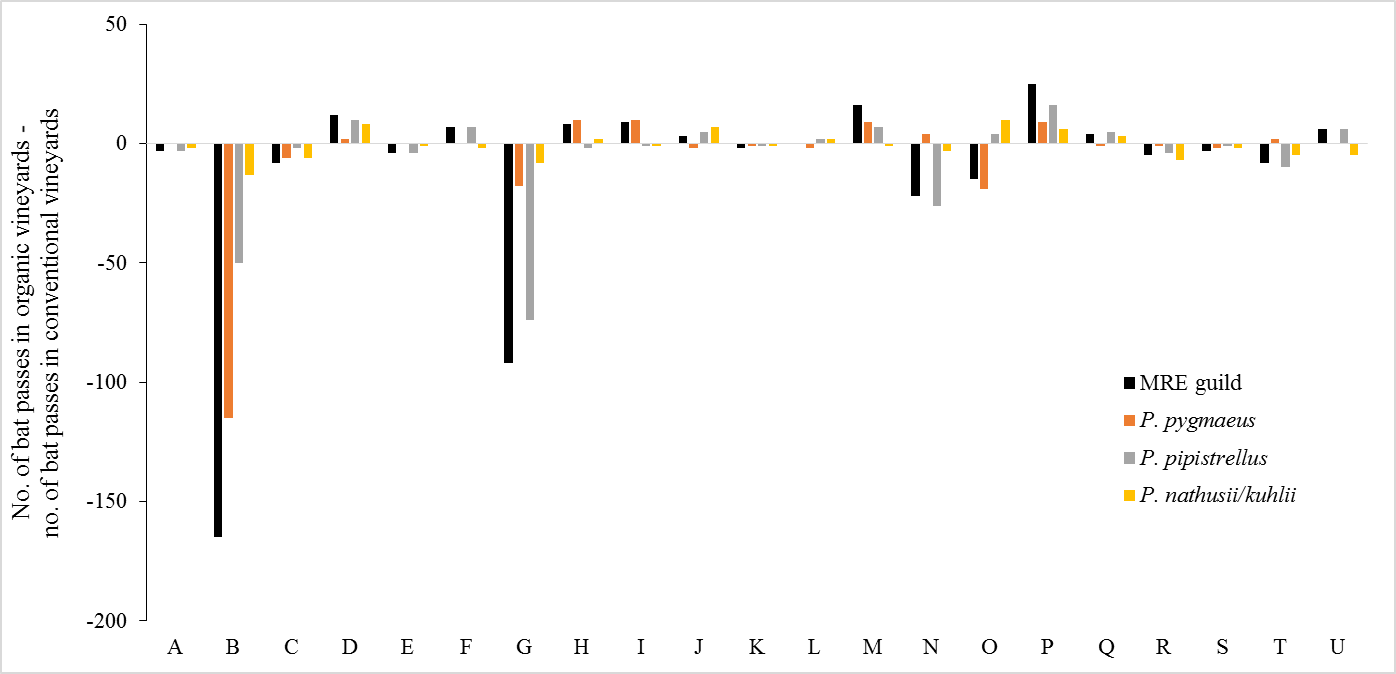


**Figure S1.** Paired-sites breakdown of the differences in bat activity (i.e., no. of bat passes) between the two farming types (organic vs. conventional vineyards). Letters represent the 21 paired-sites. Positive values indicate that bats were more active in organic vineyards, negative values denote that activity of bats was higher in conventional ones, and null values mean that activity level was identic in both vineyard types.

**References**

Nakagawa, S., Schielzeth, H., 2013. A general and simple method for obtaining *R*^2^ from generalized linear mixed-effects models. Methods in Ecology and Evolution 2, 133-142.

Wood, S.N., 2006. Generalized Additive Models: an introduction with R. Chapman & Hall/CRC, Boca Raton, USA.
